# ROS regulation in gliomas: implications for treatment strategies

**DOI:** 10.3389/fimmu.2023.1259797

**Published:** 2023-12-07

**Authors:** Yu-Chen Yang, Yu Zhu, Si-Jia Sun, Can-Jun Zhao, Yang Bai, Jin Wang, Li-Tian Ma

**Affiliations:** ^1^ Department of Traditional Chinese Medicine, Tangdu Hospital, Air Force Medical University (Fourth Military Medical University), Xi’an, China; ^2^ College of Health, Dongguan Polytechnic, Dongguan, China; ^3^ The Second Affiliated Hospital of Guangzhou University of Chinese Medicine, Guangzhou, China; ^4^ Department of Postgraduate Work, Xi’an Medical University, Xi’an, China; ^5^ Department of Neurosurgery, General Hospital of Northern Theater Command, Shenyang, China; ^6^ Department of Radiation Protection Medicine, Faculty of Preventive Medicine, Air Force Medical University (Fourth Military Medical University), Xi’an, China; ^7^ Shaanxi Key Laboratory of Free Radical and Medicine, Xi’an, China; ^8^ Key Laboratory of Integrated Traditional Chinese and Western Medicine Tumor Diagnosis and Treatment in Shaanxi Province, Xi’an, China; ^9^ Department of Gastroenterology, Tangdu Hospital, Air Force Medical University (Fourth Military Medical University), Xi’an, China

**Keywords:** glioma, ROS, tumor microenvironment, antioxidants, photodynamic therapy, sonodynamic therapy, chemodynamic therapy, nanodrug delivery platforms

## Abstract

Gliomas are one of the most common primary malignant tumours of the central nervous system (CNS), of which glioblastomas (GBMs) are the most common and destructive type. The glioma tumour microenvironment (TME) has unique characteristics, such as hypoxia, the blood-brain barrier (BBB), reactive oxygen species (ROS) and tumour neovascularization. Therefore, the traditional treatment effect is limited. As cellular oxidative metabolites, ROS not only promote the occurrence and development of gliomas but also affect immune cells in the immune microenvironment. In contrast, either too high or too low ROS levels are detrimental to the survival of glioma cells, which indicates the threshold of ROS. Therefore, an in-depth understanding of the mechanisms of ROS production and scavenging, the threshold of ROS, and the role of ROS in the glioma TME can provide new methods and strategies for glioma treatment. Current methods to increase ROS include photodynamic therapy (PDT), sonodynamic therapy (SDT), and chemodynamic therapy (CDT), etc., and methods to eliminate ROS include the ingestion of antioxidants. Increasing/scavenging ROS is potentially applicable treatment, and further studies will help to provide more effective strategies for glioma treatment.

## 1 Introduction

Gliomas are the most common primary malignant tumours of the central nervous system (CNS), accounting for approximately 30% of all primary brain and CNS tumours and 80% of malignant brain tumours (1). According to the criteria set by the World Health Organization (WHO), the malignancy of gliomas is divided into grades I-IV, ranging from mild to severe. Glioblastomas (GBMs) are grade IV gliomas and are the most common type. Unfortunately, GBMs are also the most dangerous, with relapses being inevitable even after rigorous treatment (2). Due to the unique characteristics of the glioma tumour microenvironment (TME), such as hypoxia, the blood–brain barrier (BBB), reactive oxygen species (ROS), and tumour neovascularization, treatment often show poor efficacy (3–5). The standard treatment for GBMs is resection followed by radiotherapy and temozolomide (TMZ) chemotherapy, but the median survival of GBM patients is only 14.6 months (6). In addition, the humanized IgG1 monoclonal antibody bevacizumab is also commonly used in the clinical treatment of GBMs (7). According to the available studies, neither TMZ nor bevacizumab is sufficient to treat gliomas. TMZ causes alkylation of genomic DNA at the N^7^ and O^6^ sites of guanine and at the N^3^ site of adenine. When the alkylation lesion at the guanine O^6^ position is not repaired, it leads to mispairing during DNA replication, which triggers a break in the DNA strand and causes GBM cell death (8–10). However, O^6^-methylguanine-DNA methyltransferase (MGMT) exists in GBM cells, cleans the alkyl group produced by TMZ and repairs damaged DNA. The presence of MGMT is an important reason for the resistance of GBMs to TMZ (11). Bevacizumab targets a protein called vascular endothelial growth factor-A (VEGF-A) and slows tumour growth and proliferation by preventing tumour angiogenesis, thereby depriving GBM cells of nutrient uptake (7). However, due to tumour heterogeneity and insufficient pharmacokinetics, it is still difficult to prevent GBM recurrence with antiangiogenic therapy (12–14). Therefore, the search for new treatment methods for gliomas has become a hotspot of current research.

ROS are reactive substances produced by oxygen reduction, including hydrogen peroxide (H_2_O_2_), organic hydroperoxide (ROOH), singlet oxygen (^1^O_2_), ozone (O_3_), superoxide anion (O_2^˙‾^_), hydroxyl radical (OH·), and peroxyl radical (ROO·) (15), etc. Certain levels of ROS are required for cell survival and are involved in cell proliferation and differentiation (16), skeletal muscle contraction (17), immune response (18) and other processes. These physiological effects are based on the regulation of multiple signalling pathways by ROS, such as the nuclear factor-kappaB (NF-κB), phosphatidylinositol 3-kinase (PI3K)/protein kinase B (AKT), and mitogen-activated protein kinases (MAPKs) (19, 20). The normal function of cells also depends on the ROS threshold, which represents the critical point of intracellular ROS levels (15, 21). A level of ROS slightly below the threshold is helpful to maintain normal cell function. However, when ROS persistently accumulate abnormally beyond the threshold, they may cause irreversible oxidative damage to cells or even lead to cell death (21).

In gliomas, appropriate amounts of ROS can activate growth-related signalling pathways, induce DNA mutations, and promote invasion and metastasis (22–24). However, it has been shown that inducing ROS accumulation leads to glioma cell death (25, 26). In contrast, given the critical role of ROS in the cell, the depletion of ROS also makes it difficult for glioma cells to survive (27, 28). Therefore, controlling the level of ROS becomes a potential strategy for glioma treatment. According to the literature, methods to induce massive ROS production include photodynamic therapy (PDT) (29), sonodynamic therapy (SDT) (30) and chemodynamic therapy (CDT) (31). The main approach to ROS reduction is the application of various antioxidants (27, 28, 32). All these methods have the potential to be used to treat gliomas. Therefore, we need to better understand the mechanisms of ROS production and clearance in gliomas, as well as their role in the glioma TME. Meanwhile, the methods based on ROS generation/scavenging also contribute to the prevention and treatment of gliomas.

## 2 ROS production and antioxidant defence systems

ROS production is caused by exogenous environmental stimuli or endogenous metabolism. Exogenous ROS can be generated by environmental pollutants, such as heavy metals (33), ultraviolet radiation (34), asbestos (35), sulfur dioxide (36), and particulate matter with a diameter of less than 2.5 µm (PM2.5) (37). Endogenous ROS production is mainly dependent on the mitochondrial electron transport chain (ETC) (38) and NADPH oxidases (NOXs) (39). In some cases, peroxisomes (40) and endoplasmic reticulum membranes (41) have also been identified as ROS production sites.

When ROS levels are elevated, glioma cells initiate their own antioxidant defence system in response to oxidative stress (OS). These antioxidant defence systems consist of a series of enzymes, such as superoxide dismutase (SOD) (42), catalase (CAT) (43), glutathione peroxidase (GPX) (44), glutathione reductase (GSR) (45), haem oxygenase (HMOX) (46), peroxiredoxin (PRDX) (47), thioredoxin (TRX) (48), and quinone oxidoreductase 1 (NQO1) (49). Nonenzymes include glutathione (GSH) (50), α-lipoic acid (51), and coenzyme Q10 (CoQ10) (52). Of note, nuclear factor erythroid 2-related factor 2 (NRF2) is a basic leucine zipper (bZIP) transcription factor that is an important controller of the activation of cellular antioxidant defence systems (53). Under normal conditions, Kelch-like ECH-associated protein 1 (KEAP1) can promote the polyubiquitination and degradation of NRF2 to maintain a certain level of NRF2. However, under OS, KEAP1 is oxidized, and NRF2 enters the nucleus and binds to the antioxidant response element (ARE) sequence, thereby activating the expression of the abovementioned series of antioxidant enzymes (54–58). In addition, after DNA damage caused by OS, DNA repair mechanisms in glioma cells are activated to repair damaged DNA and exert indirect antioxidant effects, such as direct repair (59–61), base excision repair (BER) (62), mismatch repair (MMR) (63, 64), and nucleotide excision repair (NER) (65, 66). A summary of the antioxidant defence systems in glioma cells is presented in **Table 1**.

**Table 1:** Glioma-associated antioxidant defence systems.

Classification	Name	Function	Related research	Reference
Antioxidant enzymes	SOD	It catalyses O_2_ **^•-^** to form molecular oxygen and H_2_O_2._	1) After treatment with the EZH2 inhibitor GSK343, the levels of SOD decrease and the production of ROS increases in GBM cells, ultimately leading to cell death. 2) After knocking down the *eIF4E* gene, there is a decrease in SOD levels and an increase in H_2_O_2_ levels in glioma cells, ultimately leading to cell death. 3) Mutation of BRG1 in gliomas leads to elevated levels of ROS and reduced expression of SOD, thereby increasing the sensitivity of gliomas to TMZ treatment. 4) After TMZ treatment, there is an increase in the expression of Sp1, an upregulation of SOD2, a reduction in ROS levels, ultimately promoting the survival of GBM cells.	(42, 67–70)
	CAT	It decomposes H_2_O_2_ generated by SOD.	1) The overexpression of CAT and reduction of H_2_O_2_ contribute to the resistance of glioma cells to TMZ. 2) Knocking down CEBPD downregulates CAT expression and increases H_2_O_2_ levels, consequently inducing GBM cell death.	(43, 71)
	GPX	They are selenium-containing enzymes that ensure the detoxification of H_2_O_2_ and lipid peroxides.	1) The curcumin analogue ALZ003 promotes AR degradation, enhances ROS levels, and inhibits GPX4, ultimately resulting in GBM cell death. 2) Plumbagin induces the degradation of GPX4 and elevates ROS levels, consequently inhibiting the growth of GBMs.	(44, 72, 73)
	GSR	It binds to NADPH and prevents oxidative damage.	Overexpression of GSR decreases ROS levels, thereby promoting glioma resistance to TMZ.	(45, 74)
	HMOX	It catalyses the formation of the antioxidant bilirubin from heme.	1) Chaetocin sensitizes GBM cells to TRAIL-induced apoptosis by inducing ROS production and DNA damage. The deficiency of HMOX1 can enhance the sensitizing effect of chaetocin on TRAIL. 2) Overexpression of NRF2 can increase HMOX1 expression, reduce ROS, reduce the cytotoxicity of carmustine, and promote glioma cell survival. 3) ATO promotes glioma cell damage and HMOX1 expression by inducing the production of ROS. Inhibitors of HMOX1 significantly increase glioma cell death and ROS generation induced by ATO.	(46, 75–77)
	PRDX	It contains cysteine residues that transmit REDOX signals.	Overexpression of PRDX4 leads to the downregulation of ROS, contributing to the promotion of GSC cell survival.	(47, 78)
	TRX	It has a reducing effect and can eliminate ROS.	The use of TRX inhibitors leads to an increase in ROS levels in GBM cells, triggering cell death.	(48, 79, 80)
	NQO1	It neutralizes ROS in the plasma membrane and prevent lipid peroxidation.	1) The drug MNPC inhibits NQO1, leading to increased ROS levels and promoting GBM cell death. 2) C/EBPβ can regulate the transcription of NQO1, neutralize ROS in GBM cells, and promote proliferation. 3) NQO1 can also bind with the substrate TSB, resulting in the significant generation of ROS, and promoting GBM cell death.	(49, 81–83)
Antioxidant nonenzymes	GSH	It contains active mercaptan groups.	1)The use of ciglitazone promotes the production of ROS, leading to a decrease in GSH levels and cell death in glioma cells. 2) SAS decreases GSH levels, induces ROS production, and promotes GBM cell death. 3) Silibinin induces autophagy in glioma, resulting in the depletion of GSH, elevation of H_2_O_2_, and BNIP3-dependent nuclear translocation of AIF, ultimately leading to glioma cell death.	(50, 84–86)
	α-lipoic acid	It has both pro-oxidation and antioxidation effects.	Activation of TRPA1 induces hypoxia and OS in gliomas, potentially resulting in apoptosis. However, α-lipoic acid has been shown to reverse these effects, thereby promoting glioma cell survival.	(51, 87)
	CoQ10	Its reduced form protects against lipid peroxidation damage.	CoQ10 reduces ROS levels and shifts the oxidative balance towards a pro-oxidant state, thereby enhancing the sensitivity of GBM cells to radiation therapy and chemotherapy.	(52, 88)
DNA repair enzymes	MGMT	It is one of the direct DNA repair proteins. It repairs DNA damage caused by OS by removing methyl groups.	The upregulation of GBP3 contributes to TMZ resistance in GBMs through the induction of NRF2 and MGMT expression.	(59–61)
	PARP1	PARP1 belongs to BER, which can recognize, cleave, and repair DNA damage caused by OS.	PARP1 plays a crucial role in the repair of DNA damage caused by ROS and facilitates the survival of GBM cells.	(62, 89)
	MSH2	MSH2 belongs to MMR, which can recognize and repair incorrectly paired nucleotides generated during DNA replication.	After TMZ treatment, MEX3A expression is increased in GBM cells, binding to MSH2 mRNA and recruiting the CCR4-NOT complex to facilitate its degradation. Consequently, this leads to reduced DNA mismatch repair activity and decreased sensitivity of GBM cells to TMZ.	(63)
	MSH6	MSH6 belongs to MMR and participates in DNA mismatch repair.	MSH6 leads to TMZ resistance in GBM, and the hypoxic TME induced by MSH6 may promote GBM metastasis through EMT and angiogenesis.	(64)
	XPC	XPC belongs to NER, which can recognize, excision and repair DNA damage caused by OS.	Nuclear translocation of XPC leads to TMZ resistance in MGMT-deficient GBMs.	(65, 66, 90)
Transcription factors	NRF2	It can lead to activation of the transcription of AREs.	1) APOC1 reduces ferroptosis in GBMs by inhibiting KEAP1, promoting NRF2 nuclear translocation, increasing HMOX1 and NQO1 expression, and downregulating ROS. 2) IR-TMZ can induce the generation of ROS, leading to the upregulation of NRF2 and promoting GBM recurrence. Blocking the activation of NRF2 can enhance the sensitivity of GBMs to chemoradiotherapy. 3) S-guanylation of KEAP1 in glioma cells is induced by 8-nitro-cGMP, which leads to the activation of NRF2. Subsequently, the expression of HMOX1 is induced, while the level of H_2_O_2_ decreases, resulting in the survival of glioma cells.	(91–94)

EZH2, enhancer of zeste homologue 2; eIF4E, eukaryotic translation initiation factor 4E; BRG1, Brahma-related gene 1; Sp1, Specificity protein 1; CEBPD, CCAAT enhancer-binding protein delta; AR, androgen receptor; TRAIL,TNF-related apoptosis-inducing ligand; ATO, arsenic trioxide; C/EBPβ, CCAAT/enhancer binding protein (C/EBP)beta; TSB, tanshindiol B; SAS, sulfasalazine; BNIP3, Bcl-2 19-kDa interacting protein 3; AIF,apoptosis inducing factor; TRPA1, transient receptor potential ankyrin 1; GBP3,guanylate binding protein 3; PARP1, poly(ADP-ribose) polymerase 1; MSH2/6, MutS homologue 2/6; MEX3A, Mex-3 RNA binding family member A; CCR4-NOT, carbon catabolite-repression 4-Not; XPC, xeroderma pigmentosum complementation group C; APOC1, apolipoprotein C1; IR, irradiation; 8-nitro-cGMP, 8-nitroguanosine 3’,5’-cyclic monophosphate.

## 3 The role of ROS in the glioma TME

**Figure 1:**
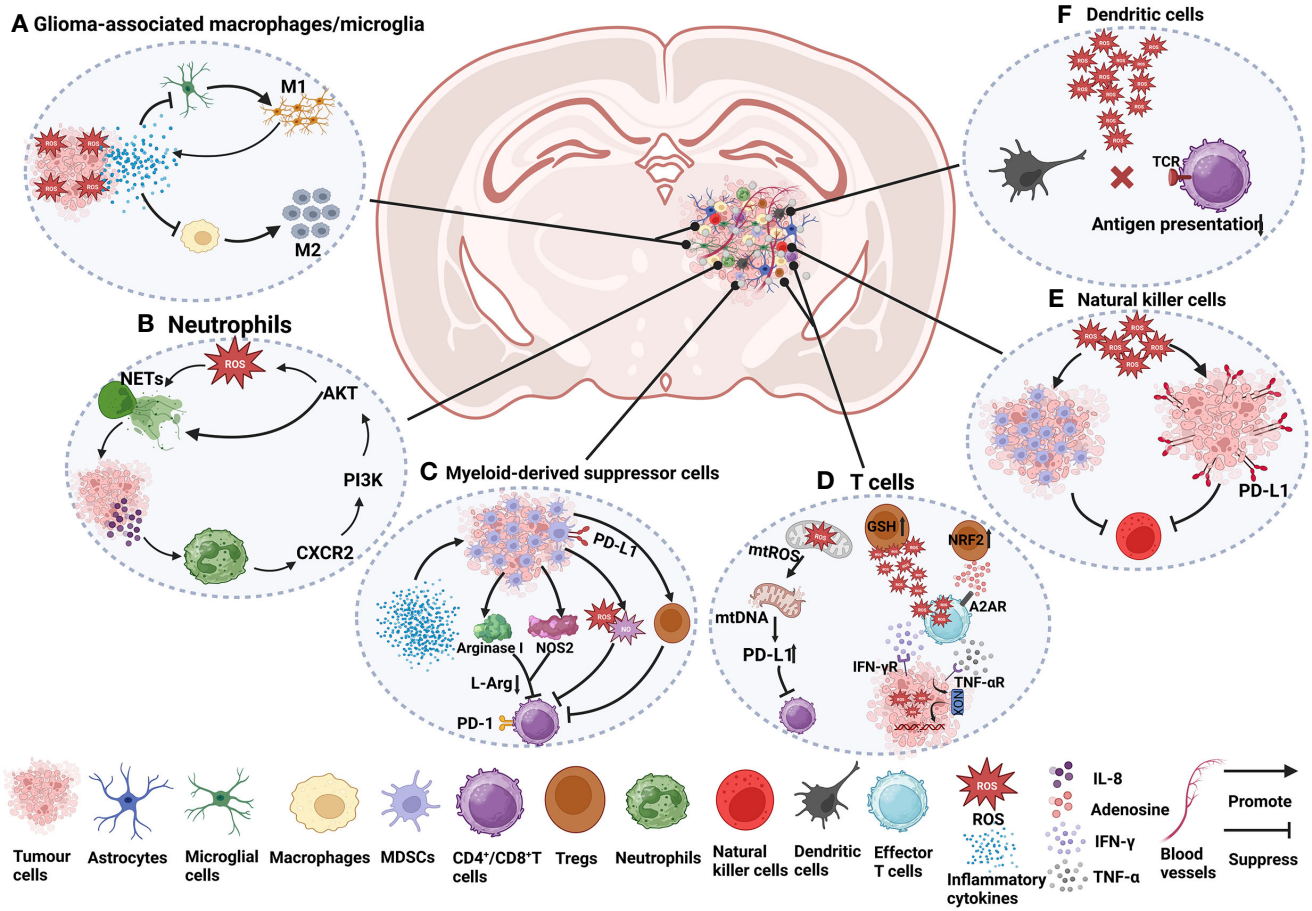
The role of ROS in immune cells in the glioma TME. **(A)** Under ROS stress, tumour cells secrete many cytokines, such as IL-4, IL-6, IL-10, and TGF-β, which cause macrophage immunosuppression and facilitate the recruitment of M2 tumour-associated macrophages (97–99). The activation of microglia is mainly manifested as the M1 type, accompanied by the release of a series of inflammatory factors (96, 100). **(B)** NETs induce glioma cells to secrete IL-8 to recruit neutrophils, promote the CXCR2/PI3K/AKT/ROS signalling axis, and finally promote the formation of NETs, forming a positive feedback pathway (101). **(C)** Cytokines and growth factors, such as granulocyte-macrophage colony stimulating factor (GM-CSF), granulocyte colony stimulating factor (G-CSF), IL-2, IL-1β, IL-6, and VEGF, can induce the aggregation of MDSCs in tumour hosts (102, 103). In the plasma of GBM patients, the level of arginase is often increased, which is related to the inhibitory function of MDSCs (102). Arginase I reduces L-arginine (L-Arg) levels (104), thereby inhibiting T-cell activation (103). Nitric oxide synthase 2 (NOS2) is another major catabolic enzyme for L-Arg metabolism in MDSCs (105). MDSCs also secrete NO and ROS, which induce T-cell inhibition (105). MDSCs also indirectly affect the activation of T cells by inducing Tregs (103). CD4^+^ effector memory T cells (CD4^+^ T_EM_) infiltrated by GBM strongly upregulate PD-1, and the corresponding ligand PD-L1 is expressed in MDSCs from tumours, which are involved in functional T-cell exhaustion (106). **(D)** The increase in mtROS causes mitochondrial DNA damage and upregulates the expression of PD-L1 to inhibit T-cell activation (97, 107). ROS produced by Tregs can suppress effector T cells (CD4^+^ and CD8^+^). Effector T cells can induce an increase in ROS in tumour cells through IFN-γ and TNF-α, which can damage tumour cell DNA and lead to tumour cell death. Tregs themselves are more resistant to oxidative stress due to the increased activity of the antioxidant system, for example, by increasing GSH and upregulating NRF2 (108). Adenosine produced by Tregs can also inhibit effector T-cell function in an A2AR-dependent manner (109). **(E)** ROS induce the proliferation of MDSCs and inhibit NK cell function (110). In addition, high levels of ROS promote PD-L1 expression on cancer cells, thereby inactivating NK cells (111). **(F)** High levels of ROS can disrupt antigen presentation between T cells and DCs, which in turn affects the recognition of tumour antigens by T cells (98, 108).

The glioma TME plays an important role in the growth, invasion, recurrence and drug resistance of gliomas. Its major components include glioma cells, immune cells, signalling molecules, stromal cells, and extracellular matrix (ECM) (95). Immune cells include glioma-associated macrophages/microglia (GAMs), myeloid-derived suppressor cells (MDSCs), T cells, monocytes, neutrophils, dendritic cells (DCs) and natural killer (NK) cells. Signalling molecules include chemokines, cytokines, growth factors, and angiogenesis factors. Stromal cells include astrocytes and endothelial cells. The extracellular matrix (ECM) is a three-dimensional structure composed of fibrin, proteoglycans and other molecules that provides biochemical and structural support for surrounding cells and plays an important role in glioma invasion and metastasis (95, 96). Among them, as important regulatory molecules, the presence of ROS have a significant impact on the glioma TME. ROS not only affect the function of immune cells (**Figure 1**) but also participate in the process of glioma cell proliferation, invasion, metastasis and death. These studies will be discussed in this section.

### 3.1 GAMs

Macrophages and microglia are important cell types in the immune system. Macrophages are primarily derived from bone marrow-resident haematopoietic stem cells (112). After entering the blood, mature mononuclear macrophages can settle in different tissues, such as the liver, lung, brain, lymph nodes and other organs and tissues, at which time they will become macrophages. Macrophages are involved in phagocytosis and clearance of pathogenic microorganisms, necrotic tissues, and secretion of a variety of inflammatory mediators involved in immune regulation and tissue repair (113–115). Microglia are induced by the colony-stimulating factor 1 receptor (CSF1R) and are generated from red myeloid progenitors of the yolk sac. They are self-renewing and reside in the CNS for a long time (116). Microglia play an important role in maintaining the homeostasis of the nervous system, including phagocytosis (117), promoting synapse formation (118), and supplying nutrients (119).

In the TME, macrophages can manifest as the M1 type (characterized by inflammatory and antitumour responses) or M2 type (involved in the repair of damaged tissues and anti-inflammation), but the TME tends to induce the differentiation of macrophages towards the M2 type (120–122). Anti-inflammatory factors released by tumours, such as interleukin (IL)-4, IL-10, and transforming growth factor-β (TGF-β), can promote the transformation of macrophages into the M2 type (123). M2 macrophages similarly release growth factors, such as vascular endothelial growth factor (VEGF), epidermal growth factor (EGF), and fibroblast growth factor (FGF), which promote tumour cell proliferation, invasion, and metastasis (124).

Glioma-infiltrated macrophages and microglia are collectively referred to as GAMs, which represent the largest population of cells infiltrating tumours, accounting for more than 1/3 of the total tumour mass (125, 126). GAMs play an important role in the glioma TME and promote tumour progression. First, anti-inflammatory factors such as IL-10 and TGF-β produced by GAMs inhibit the function of other immune cells in the TME and weaken the antitumour immune response (127, 128). Second, GAMs also secrete matrix metallopeptidase 2 (MMP2) and MMP9, which are able to breakdown matrix proteins, such as collagen and fibronectin, thereby enabling glioma cells to penetrate and invade the surrounding stromal tissues (129). Finally, GAMs also secrete proangiogenic molecules, such as VEGF and CXC motif chemokine ligand 2 (CXCL2), which have been shown to promote glioma growth and metastasis (129, 130).

In the glioma TME, ROS generally induce the generation of M2 GAMs (96, 131). ROS modulator 1 (Romo1), a membrane protein located on mitochondria, was found to regulate mitochondrial ROS (mtROS) production in GBMs (132). In GBM mouse models, overexpression of Romo1 induces ROS generation via mTORC1 signalling, which in turn promotes the polarization of bone marrow-derived macrophages (BMDMs) to the M2 type, resulting in a significant suppressive TME (133). In addition, a prognostic model of human GBMs and ROS showed that high expression of ROS-related genes such as *HSPB1, LSP1* and *PTX3* was closely associated with M2 macrophages and correlated with shorter survival of GBM patients. This suggests that ROS-related genes may be potential targets for GBM treatment. Therefore, inhibiting the polarization of macrophages towards M2 type and promoting the polarization towards M1 type may be beneficial for the treatment of GBMs (134). Besides, GAMs could survive in a high ROS environment mainly due to the action of antioxidant enzymes. A study of GBM tissues in humans and mice showed that the active antioxidant enzyme GPX1 was expressed at higher levels in GAMs than in GBM cells, resulting in GAMs being able to survive in a high ROS environment (135). It is known that GPX1 plays an important role in H_2_O_2_ detoxification (136). In summary, the antioxidant enzymes in GAMs protect them from ROS damage, which is necessary for the formation of M2 GAMs.

### 3.2 MDSCs

MDSCs, which are mainly differentiated from haematopoietic stem cells in the bone marrow, are a group of myeloid cells with heterogeneous and immature characteristics (137). In normal organisms, the levels of MDSCs in the peripheral blood tend to be very low (138). MDSCs have certain immunomodulatory effects, which can regulate the inflammatory response, inhibit overactivated immune cells, prevent excessive immune responses, and reduce tissue damage (105, 139).

Upon tumour stimulation, MDSCs are activated and released into peripheral blood and tissues. However, MDSCs often suppress the immune response and cause tumour escape (140, 141). The suppressive effect of MDSCs is mainly manifested by inhibiting the activity of other immune cells, including macrophages (142), CD4^+^ T cells (106), CD8^+^ T cells (143), NK cells (144), and DCs (145). First, MDSCs can highly express arginase-1 (ARG-1), which can convert arginine to uric acid and ornithine, thereby reducing the concentration of arginine in the internal environment (105). Arginine deficiency leads to limited activation of immune cells such as T cells (146) and NK cells (147), thereby impairing the immune response and promoting tumour development and metastasis. Second, MDSCs can secrete immunosuppressive cytokines, such as TGF-β and IL-10, which can inhibit the secretion of IL-12 by macrophages, thereby blocking the activity of cytotoxic T lymphocytes (CTLs) (148, 149). TGFβ-1 secreted by MDSCs also promotes the transformation of CD4^+^ T cells into immunosuppressive Tregs (150). Finally, MDSCs can also express immunosuppressive ligands, such as programmed death ligand-1 (PD-L1), which in turn suppresses T-cell priming and activation (151).

In gliomas, MDSCs comprise approximately 30% to 50% of the tumour entity (152). The increase in MDSCs is thought to be associated with glioma progression and immune escape (153). Generally, MDSCs can be divided into two main subsets based on their phenotype and function: monocytic (mMDSCs) and granulocyte/polymorphonuclear (gMDSCs) (154). Specifically, mMDSCs represent the major subset in the GBM TME. mMDSCs in the GBM TME of humans and mice expressed higher levels of adhesion molecules, such as integrin β1 and dipeptidyl peptidase 4 (DPP4), leading to enhanced cell adhesion and further promoting tumour migration and invasion (155). In addition, MDSCs can promote angiogenesis through the release of VEGF (156), as well as the release of cytokines such as IL-10, IL-6, and TGF-β under hypoxic conditions (157), thereby promoting glioma growth and invasion.

In the TME, MDSCs can survive in a high ROS environment because of their high expression of NRF2. On the one hand, NRF2 upregulated anti-OS genes in MDSCs and protected MDSCs from OS damage. On the other hand, NRF2 enhances the immunosuppressive activity of MDSCs by increasing their ability to produce ROS (158). In gliomas, ROS in MDSCs play an important role in maintaining the function of MDSCs (159). ROS can prevent the differentiation of MDSCs and promote the formation of an immunosuppressive TME (160). Specifically, ROS maintain the undifferentiated state of MDSCs by inhibiting the differentiation of MDSCs into mature immune cells such as macrophages and DCs (145, 161, 162). This undifferentiated state allows MDSCs to continue expressing immunosuppressive molecules such as TGF-β, IL-10 and PD-L1 (163). In addition to being able to impair the antigen presentation capacity of DCs (164–166), these immunosuppressive molecules can also inhibit the activity of T cells and induce the differentiation of T cells into Tregs (141, 167). Collectively, high levels of ROS play an important role in maintaining the undifferentiated state of MDSCs, which in turn mediates the immunosuppressive TME. Therefore, targeting MDSCs may become a promising therapeutic strategy.

### 3.3 T cells

T cells are members of the adaptive immune system that respond to antigens presented by antigen-presenting cells such as DCs and macrophages (168). T cells can be divided into CD4^+^ T cells and CD8^+^ T cells based on surface markers and function (169). CD4^+^ T cells have antigen receptors on their surface, which can recognize antigen fragments presented by MHC class II molecules on the surface of antigen-presenting cells and exert immune functions by activating other types of immune cells. CD8^+^ T cells generally refer to CTLs that can directly kill infected cells by MHC class I molecules (170). Regulatory T cells (Tregs) are a subset of CD4^+^ T cells that express the transcription factor forkhead box protein 3 (FOXP3) and play a role in inhibiting pathological immune responses and maintaining homeostasis in the body (171).

In tumours, CD4^+^ T cells mainly activate other immune cells, such as CD8^+^ T cells and NK cells, to enhance the immune response (172). CD4^+^ T cells can secrete cytokines, such as interferon-gamma (IFN-γ) and tumour necrosis factor-alpha (TNF-α), which directly kills tumour cells (173). CD8^+^ T cells carry specific T-cell antigen receptors (TCRs) that recognize and bind to tumour cells expressing specific antigens, thereby releasing cytotoxins, such as perforin and granzyme, to directly kill tumour cells (174). In addition, CD8^+^ T cells can also secrete cytokines, such as IFN-γ and TNF-α, which directly inhibit tumour cell growth and proliferation (175). Tregs play an indispensable role in maintaining the homeostasis of the immune system. Tregs suppress other immune cells and prevent excessive immune responses by producing inhibitory cytokines such as IL-10, IL-35, and TGF-β. However, overactive Tregs in turn limit the antitumour ability of immune cells (176).

In gliomas, T-cell dysfunction is often caused by the strong immune escape ability of glioma cells. Some studies have shown that human GBM cells are capable of producing the immunosuppressive factor TGF-β (177), which inhibits T-cell activation, thereby weakening the immune response (178). In addition, the human glioma TME has many immunosuppressive cells, such as M2 macrophages and Tregs, whose presence usually inhibits the activity and function of T cells and is associated with reduced overall survival of patients (179–181). Moreover, human glioma cells often express immune escape sites on the surface, such as PD-L1 and B7 homologue 3 (B7-H3), which can bind to immune checkpoint receptors, thereby inhibiting the activity and function of T cells (182).

ROS play an important role in regulating T-cell function and activity. Low levels of ROS can promote the activation and proliferation of T cells to enhance immune responses (183). However, higher levels of ROS can inhibit the secretion of cytokines by T cells and induce apoptosis (184). In the TME, excessive ROS may induce apoptosis of T cells, leading to decreased antitumour ability. For example, ROS produced by neutrophils or tumour cells can be transferred to T cells and cause OS, thereby causing hyporeactivity of T cells in cancer patients (185). In mouse glioma models, the administration of hyperbaric oxygen (HBO) can induce the generation of ROS in the thymus, which subsequently inhibits the generation of CD3^+^ T cells and promotes glioma growth *in vivo* (186). Therefore, high levels of ROS in the TME may lead to impaired T-cell function, which in turn enhances tumour escape. Targeting ROS in the TME to enhance the killing ability of T cells may be a potential therapeutic option.

### 3.4 Neutrophils

Neutrophils are derived from haematopoietic stem cells in the bone marrow through differentiation and maturation. When neutrophils mature, they enter the circulation and are distributed throughout the body through the blood (187, 188). Neutrophils are important immune cells of the body that are capable of engulfing and eliminating pathogens, such as bacteria and viruses (189, 190). In addition, when tissues are injured or infected, neutrophils rapidly migrate to the damaged site and release cytokines and chemokines to trigger local inflammation (191).

The role of neutrophils in tumours is complex. On the one hand, neutrophils are capable of killing tumour cells by releasing cytotoxic ROS (192) and by direct cell contact (193). On the other hand, neutrophils also promote tumour growth and metastasis by secreting immunosuppressive factors such as TGF-β, IL-6 and IL-8 (194) and interacting with circulating tumour cells (195). Furthermore, neutrophil extracellular traps play an important role in tumour progression. In the early stages of tumour invasion and metastasis, neutrophils can release neutrophil extracellular traps (NETs), which include DNA, tissue proteins and other substances. NETs can form channels suitable for tumour cell migration and protect tumour cells from immune system attack (196, 197).

Neutrophils, as mediators of inflammation, are early markers of GBM progression (198). Overall, neutrophils promote tumour growth, invasion, and angiogenesis. Neutrophils contribute to glioma infiltration by secreting elastase (199). Furthermore, neutrophils may also become resistant to antineoplastic therapy. In patients receiving anti-VEGF therapy, neutrophils contribute to glioma resistance to anti-VEGF therapy by increasing S100A4 expression and angiogenesis in glioma tissues (200). S100A4 is known to be a biomarker expressed in glioma stem-like cells (GSCs) that induces the tumorigenic activity of neutrophils (201). In addition, some studies have shown that the expression of MDSCs is increased in the peripheral blood of GBM patients, of which the neutrophilic MDSC subset accounts for the largest proportion, accounting for approximately 60% (102). Neutrophilic MDSCs derived from the peripheral blood of GBM patients can inhibit T-cell proliferation *in vitro*, which is related to the high expression of PD-L1 on effector memory CD4^+^ T cells (106).

Several studies have shown that ROS are important factors in promoting the formation of NETs (202–204). In chronic granulomas, NOXs are activated by protein kinase C (PKC) and produces ROS. These ROS can act as signalling molecules, causing neutrophils to release DNA and form a mesh-like structure, which then combines with the adhered granule proteins to form NETs (202). Furthermore, in primary mouse and human neutrophils, members of the MAPK family, such as c-Jun N-terminal kinases (JNKs) (203), extracellular signal-regulated kinases (ERKs) and p38 (204), can activate NOXs to generate ROS, which in turn induces the production of NETs. Similarly, ROS are similarly closely related to NETs in GBM TME. A study in human GBMs showed that NETs promote IL-8 secretion in GBMs by stimulating the NF-κB signalling pathway, which in turn stimulates endothelial cells to generate blood vessels to deliver essential nutrients and oxygen to the tumour site (101). When IL-8 binds to C-X-C motif receptor 2 (CXCR2) on neutrophils, it mediates the formation of NETs through the CXCR2/PI3K/AKT/ROS axis. This positive feedback loop stimulates the interaction between NETs and GBM cells and leads to profound changes in the TME (101). Recent studies in murine models of GBMs have additionally demonstrated that neutrophils promote the necrosis of GBM cells by transferring particles containing myeloperoxidase into these cells. This phenomenon induces OS, which is a result of the iron-dependent accumulation of lipid peroxides in GBM cells (205).

### 3.5 DCs

DCs, which differentiate from bone marrow haematopoietic stem cells through common DC progenitors (CDPs), play an important immunomodulatory role by presenting antigens (206). In tissues, DCs are usually naturally present and are considered to serve as a bridge connecting innate and adaptive immunity and are able to promote the transformation of innate to adaptive immune responses (207). Innate immunity refers to the immunity possessed by individuals at birth, which has a wide range of effects and is not triggered by specific antigens (208). Adaptive immunity is mainly the ability to respond to and adapt to a specific antigen or pathogen, which is achieved through T-cell-mediated cellular immunity and antibody-mediated humoral immunity (209).

Tumour formation is often accompanied by the expression of tumour antigens. Tumour antigens can be captured and processed by DCs and subsequently presented to naive T cells to induce their proliferation and differentiation into effector cells, such as CD8^+^ T cells, which subsequently kill the tumours (210). Furthermore, DCs can produce a variety of immune stimulating factors, such as cytokines and chemokines, which induce DCs and NK cells to reach inflammatory sites (211), as well as induce the activation of tumour-specific T cells (212).

Antigens released by glioma cells can be captured and processed by DCs, presented to T cells, and activate effector T-cell function (213). However, glioma cells can often evade immune surveillance by inhibiting the maturation of DCs. Studies have shown that tumour-conditioned medium (TCM) collected from the supernatant of human primary glioma cells can upregulate the expression of suppressor of cytokine signalling 1 (SOCS1) in DCs and then inhibit the NF-κB signalling pathway, thereby limiting the maturation of DCs. The subsequent suppression of T-cell activity, as well as IFN-γ secretion, results in immune escape of glioma cells (214). A previous study revealed that mouse gliomas have the ability to secrete cell factors including TGF-β and IL-10 (215). These factors are known to impede the maturation and functionality of DCs within the TME (216).

In addition, recent studies on the role of DCs in the progression of human GBMs have focused on the maintenance of DC homeostasis. Overexpression of NRF2 in DCs leads to the inhibition of DC maturation and subsequently reduces effector T-cell activation, which may be related to the decrease in ROS levels mediated by NRF2. In contrast, inhibition of NRF2 promotes the maturation of CD80^+^ and CD86^+^ DCs (217).

### 3.6 NK cells

NK cells belong to a type of lymphocyte that can eliminate tumour cells without specific antigens and are an important part of innate immunity (218). NK cells are derived from bone marrow haematopoietic stem cells and enter the circulation after maturation (219). Approximately 5-15% of lymphocytes in normal blood are NK cells (220).

NK cells have the ability to kill tumour cells. First, NK cells kill tumour cells by making direct contact with tumour cells that express specific ligands. There are a variety of activated receptors on the surface of NK cells, such as natural killer group 2 member D (NKG2D) and natural cytotoxicity receptors (NCRs; NKp46, NKp44 and NKp30) (221), etc. Among them, NKG2D is one of the most studied receptors and is able to recognize ligands on the surface of tumour cells, such as major histocompatibility complex class I polypeptide-related sequence A and B (MICA/B) and UL16-binding protein (ULBP). This recognition activates NK cells and prompts them to kill tumour cells (222). Second, NK cells can kill tumour cells through the antibody-dependent cellular cytotoxicity (ADCC) mechanism. When the antigens on the surface of tumour cells are labelled with specific antibodies, NK cells can bind to the specific antibodies through the CD16 (FcγRIIIa) receptor on their surface. Activated NK cells then release particles containing perforin and granzyme, which trigger apoptosis of antibody-labelled tumour cells (223, 224). Furthermore, NK cells can produce cytokines, such as IFN-γ and TNF-α, which can enter tumour cells and thus kill them (225). Moreover, IFN-γ released by NK cells can also inhibit tumour angiogenesis, thereby impeding tumour nutrient supply (226).

NK cells also have a killing effect on gliomas. First, NK cells can kill glioma cells by secreting perforin and granzyme B upon induction by IFN-β (227). Second, NK cells kill gliomas by specific activating receptors on their surface. When NKG2D and DNAX accessory molecule-1 (DNAM-1) on the surface of human NK cells bind to their ligands on the surface of GBM cells, they can trigger NK cell cytotoxicity and cause GBM cell death (228, 229). However, human GBM-derived TGF-β may lead to downregulation of NKG2D receptors on the surface of NK cells and contribute to GBM cell survival, suggesting that blocking TGF-β may be beneficial in the treatment of GBMs (230). Similarly, NK cells can also kill GBM cells through ADCC. Cetuximab is a monoclonal antibody that binds epidermal growth factor receptor (EGFR) on tumour cells (231). When administered, cetuximab binds to a human GBM surface antigen (EGFRvIII) and activates fragment crystallizable (Fc) receptors on NK cells, leading to NK cell-mediated cytotoxicity against GBM cells (232).

NK cells are often particularly sensitive to the cytotoxic effects of ROS, and their antitumour activity is often inhibited by ROS in the solid tumour TME, whereas antioxidant therapy may partially restore NK cell function (110, 233). Previous studies have shown that high levels of ROS in rats with fibrosarcoma can limit the adhesion of NK cells to similarly charged tumour cells by promoting the accumulation of anionic charges on their surface. This disadvantage can be prevented by antioxidant molecules such as CAT and SOD (234). Similarly, *in vitro*, the CD20 antibody rituximab triggered monocyte ROS production, which in turn inhibited the ADCC effect of NK cells on human primary leukaemia cells. However, antioxidant treatment (histamine dihydrochloride and diphenylene iodonium chloride) partially restored the ADCC effect of NK cells (235). At present, although some studies have suggested the inhibitory effect of ROS on NK cell activity in the TME, the study of ROS in NK cells in the glioma TME is still limited. Further studies will help to understand the effect of ROS on NK cell function in the glioma TME.

### 3.7 Glioma cells

The threshold of ROS is very important in cancer therapy. When ROS produced by tumour cells exceed a certain threshold and cannot be detoxified by antioxidants, it results in high levels of OS, which drives cancer cell death or cause them to become more sensitive to treatment. However, a low level of ROS in tumour cells contributes to their growth, proliferation, invasion and metastasis (236). Therefore, tumour cells need to maintain their ROS levels to maintain their survival and invasive abilities (237).

A study of tumour tissues and blood samples from glioma patients found that abnormal increases in ROS caused DNA damage in glioma cells, resulting in high expression of the DNA damage marker 8-oxo-deoxyguanosine (8-oxo-dG) and low expression of the epigenetic marker 5-methylcytosine (m5C). This is associated with increased malignancy of gliomas (22). In mouse models, glioma cells can overexpress aquaporin 8 (AQP8), which increases ROS levels, resulting in decreased expression of phosphatase and tensin homolog (PTEN) and increased expression of phosphorylated (p)-AKT, thereby promoting the growth and proliferation of gliomas (23). Moreover, the production of a significant amount of ROS induced by 12-O-tetradecanoylphorbol-13-acetate (TPA) can activate the MAPK pathway and cyclooxygenase-2 (COX-2)/prostaglandin E2 (PGE2) pathways, subsequently enhancing the *in vitro* migration and invasion capability of glioma cells (24). In contrast, high levels of ROS activate regulated glioma cell death programs, including apoptosis, necrosis, autophagy, ferroptosis, etc. For example, *in vitro*, salinomycin can activate p53, trigger the opening of mitochondrial permeability transition pore (mPTP), and induce the production and accumulation of mtROS, leading to the necrosis of glioma cells (25). The activation of transient receptor potential mucolipin 1 (TRPML1) inhibits autophagy in glioma cells *in vitro*, leading to ROS production and subsequent induction of apoptosis (238). Similarly, the increase in ROS induced by isoaaptamine also leads to apoptosis and autophagy in GBM cells *in vitro* (26). Furthermore, the heat shock protein 90 (HSP90) and dynamin-related protein 1 (DRP1) increase ROS production by regulating the expression of acyl-coenzyme A synthetase long-chain family member 4 (ACSL4) and mitochondrial morphology, leading to ferroptosis of gliomas in mice *in vitro* and *in vivo* (239).

Glioma stem-like cells (GSCs) are a subpopulation of GBM cells with stem cell characteristics. They have self-renewal, tumourigenicity and multidirectional differentiation potential and are closely related to the occurrence, development, treatment resistance and recurrence of GBMs (240). Many studies have confirmed that ROS are involved in the proliferation, self-renewal and differentiation of GSCs (241, 242). In a study conducted on human-derived GSCs, it was found that TGF-β upregulated the expression of the *NOX4* gene, leading to the generation of ROS. Consequently, this ROS generation promoted GSC proliferation and maintained their stem cell state (241). Other studies have shown that serum stimulation in an *in vitro* environment is able to cause an increase in mitochondrial ROS within GSCs and modulate differentiation signalling pathways in GSCs. Interestingly, in the *in vivo* environment, increased ROS could greatly enhance glioma formation, which may be related to the activation of the NF-κB pathway by ROS (243). Compared with other tumour cells, GSCs have stronger antioxidant capacity (47). *In vitro*, the highly expressed antioxidant protein PRDX4 was able to mitigate OS in GSCs by reducing ROS generated by the protein folding process (47, 244). Furthermore, GSCs also inhibit mitochondrial respiration by increasing the expression of mitochondrial uncoupling protein 2 (UCP2), thereby alleviating OS caused by high levels of intracellular ROS and ensuring their own survival (245).

However, the understanding of the ROS threshold in cancer cells is still unclear. Measurement of the ROS threshold requires the consideration of multiple factors, including the concentration and type of ROS, the activity of intracellular antioxidant enzymes, and the type and physiological state of tumour cells (246–249). Therefore, more studies are needed to fully assess ROS thresholds and determine their impact on tumour cells. Overall, both ROS and thresholds play a crucial role in glioma cells. This provides a new research direction for ROS-based glioma therapy.

## 4 ROS-based glioma therapy

High levels of ROS are usually present in the glioma TME. On the one hand, these ROS are involved in the formation of a suppressive TME. On the other hand, they are involved in the process of glioma proliferation, invasion and migration. However, there is also a threshold for the levels of ROS in glioma cells. The induction of ROS production above the threshold can lead to an excessive OS response, causing DNA and protein damage and leading to glioma cell death (250). Conversely, depletion of ROS may also lead to the blocking of important signalling pathways involved in ROS, thereby promoting glioma death (27, 28, 32). Based on these findings, it is suggested that both methods of inducing ROS production and ROS scavenging have potential in the glioma treatment. These two therapeutic strategies may help to suppress glioma growth, enhance the immune response and improve the efficacy of other antitumour therapies.

### 4.1 Treatment to increase ROS levels

Excessive ROS can induce tumour death, so amplifying the effect of ROS may be a good way to kill tumours. For example, the use of PDT (251), SDT (252), CDT (253), can be beneficial therapies for GBMs. This part mainly summarizes the research progress of PDT, SDT and CDT in the treatment of gliomas, and discusses the application of nanodrug delivery platforms in them.

#### 4.1.1 PDT

PDT is a technique that relies on ROS production to treat nononcological diseases as well as tumours. Its main components are excitation light, photosensitizers (PSs) and ROS (254). PSs are important components in determining the efficacy of PDT (255). Photoactivated PSs can produce cytotoxic ROS in the presence of oxygen, resulting in the killing of target cells (256, 257). To date, numerous PSs have been applied in the studies of gliomas, such as 5-aminolevulinic acid (5-ALA) (258), boronated porphyrin (BOPP) (259), talaporfin (260), and temoporfin (261). The wavelength of the light is also important. The optimal PDT wavelength is between 650 and 850 nm and should be consistent with the longest wavelength absorption band of the PSs, that is, the wavelength range corresponding to sufficient energy for maximum tissue penetration to result in sufficient ROS production (262). Notably, ROS produced by PSs, such as ^1^O_2_, O_2^˙‾^_, OH, OOH·and H_2_O_2_, are essential for killing tumours. The formation of O_2^˙‾^_ and free radicals is called a type I reaction, and the formation of ^1^O_2_ is called a type II reaction (263, 264).

PDT has been approved by the United States Food and Drug Administration (FDA) for the treatment of a variety of cancers, including skin cancer, oesophageal cancer, and lung cancer (265–267). PDT has been studied since the 1980s (268, 269), and has shown promising efficacy in many glioma preclinical studies (270–272). A bibliometric analysis of literature in the field of cancer PDT (CPDT) reveals that research on CPDT is showing a rapid growth trend over the past 20 years. Among them, nanotechnology-based PDT and enhanced PDT are the current research hotspots (273). However, PDT has still not been widely adopted due to its potential toxicity to healthy brain tissues, limited light penetration, and poor targeting (251, 274, 275).

In the past 10 years, three promising phase I/II clinical trials of PDT for glioma treatment have been conducted in adults and one has been conducted in minors. A total of four clinical trials were conducted for three drugs (photofrin, ALA, photobac^®^) (**Table 2**). Among them, NCT01682746 included 5 adolescent patients with brain tumours. The incidence of serious adverse events within 1 month of PDT treatment, the progression-free survival and the overall survival within 3 years were recorded, but no results of this clinical trial were reported. NCT03048240 included 10 adult patients with newly diagnosed GBM who were treated with 5-ALA fluorescence-guided surgery followed by intraoperative PDT (based on clinicaltrials.gov). At the interim analysis, the median progression-free survival (mPFS) was 17.1 months, and the median overall survival (mOS) was 23.1 months (276). This clinical trial result indicates that intraoperative PDT is a good option for treating recurrent gliomas.

**Table 2:** Summary of completed/ongoing phase I/II clinical trials of ROS-generated PDT/SDT for glioma treatment by July 2023 (based on clinicaltrials.gov).

	NCT Number	Phases	Conditions	Interventions	Age	Enrolment	Primary endpoint and duration	Status	Related publications
**PDT**	NCT01682746	Phase 1	Brain Tumour	Photofrin photodynamic therapy	6 months to 18 years	5	More than 33% of subjects experienced neurotoxicity, photosensitivity, ocular sensitivity, or other toxicities greater than or equal to CTCAE grade 4 (4 weeks).	Completed in 2018	_____
	NCT03048240	Not Applicable	GBM	5-ALA photodynamic therapy	≥18 years	10	At least 6/10 patients benefited without unacceptable and unexpected toxicities (4 weeks).	Completed in 2021	(276)
	NCT04391062	Phase 2	GBM	5-ALA photodynamic therapy	≥18 years	21	Dose level above which dose-limiting toxicities is observed in more than 33%of subjects in an arm (4 weeks).	Enrolment ongoing	_____
	NCT05363826	Phase 1	GBM or Gliosarcoma	Photobac^®^ photodynamic therapy	≥18 years	30	Toxicity (24 hours); MTD (1 week); Photobac^®^ concentration in tumour tissues (1 hour); Photobac^®^ concentration in blood (12 weeks); PFS (18 months); OS (18 months)	Enrolment ongoing	_____
	NCT04469699	Phase 2	GBM Multiforme	Drug: Stereotactic biopsy followed by stereotactical photodynamic therapy with 5-aminolevulinic acid	18 years to 75 years	106	PFS (at least 1.5 years and a maximum of 5 years) or until progression or death	Enrolment ongoing	_____
	NCT03897491	Phase 2	GBM	5-aminolevulinic acid powder for oral solution	18 years to 70 years	20	To determine the incidence of treatment-emergent Adverse Events (2 weeks)	Enrolment ongoing	_____
**SDT**	NCT04559685	Early phase 1	High Grade Glioma	Sonodynamic therapy with MRg-FUS combined with ALA	≥18 years	30	Biological changes; Imaging changes before and after surgery (14 days)	Enrolment ongoing	_____
	NCT05362409	Phase 1	High Grade Glioma	Sonodynamic therapy with 5-ALA combined with CV01-delivered ultrasound	≥18 years	48	Incidence of adverse events; To determine the MTDu (12 months)	Enrolment ongoing	_____
	NCT05123534	Phase1/Phase2	Glioma	Sonodynamic therapy with MRg-FUS combined with ALA	≥5 years	27	Safety and Tolerability of SDT (12 months); MTD (3 weeks); Recommended Phase 2 Dose (3 weeks)	Enrolment ongoing	_____

CTCAE, Common Terminology Criteria for Adverse Events; MTD, Maximum tolerated dose; MRg-FUS, MR-Guided Focused Ultrasound device; MTDu, Maximum Tolerable Duration.

**Figure 2:**
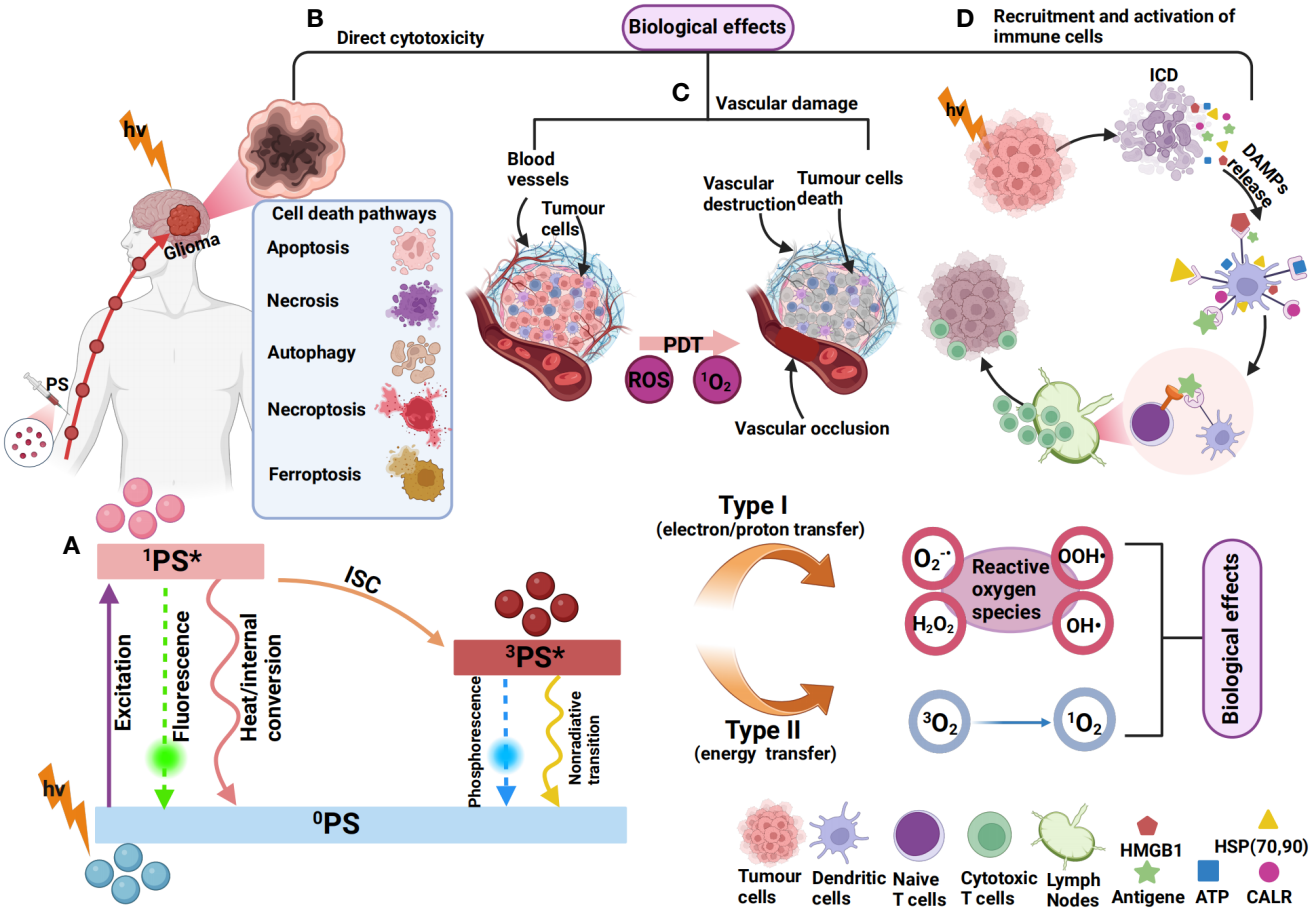
Mechanism of photodynamic immunotherapy for gliomas. **(A)** ^0^PS is activated to a singlet state (^1^PS*) after absorbing photons (hv). ^1^PS* can lose energy through internal conversion to heat and fluorescence. ^1^PS* can form a triplet state (^3^PS*) through the intersystem crossing process (ISC). ^3^PS* can be restored to 0PS by emitting phosphorescence and can also react with neighbouring molecules in two types of reactions (type I and type II). In type I reactions, ^3^PS* transfers an electron or a proton to form organic free radicals (O^2-^, OOH·, H_2_O_2_, OH·, etc.) that interact with cellular oxygen to produce cytotoxic ROS. In a type II reaction, the energy of ^3^PS* can be directly transferred to molecular oxygen (^3^O_2_) to form singlet oxygen (^1^O_2_). This results in various biological effects (264, 282, 283). **(B)** The most common types of cell death induced by PDT include apoptosis (284), autophagy (285), necrosis (286), necroptosis (287), and ferroptosis (270). **(C)** ROS produced by PDT can also cause vascular occlusion, leading to vascular damage, thereby affecting the blood supply of tumour cells (288). **(D)** PSs can induce immunogenic cell death (ICD), resulting in the exposure and release of DAMPs, such as ATP, HMGB1, CALR, HSP70/90, etc (262). The released DAMPs promote DC recruitment and maturation and present tumour antigens to T cells, leading to the activation of CD8^+^ T cells, which subsequently migrate *in vivo* to kill tumour cells (264).

The tumour killing mechanisms of PDT are various, including inducing immunogenic cell death (ICD), destroying tumour blood vessels and inducing the release of inflammatory mediators in addition to the direct killing caused by high ROS. The combination of PDT and subsequent immune response induced by PDT is referred to as photodynamic immunotherapy (PDIT) (262, 277, 278). ICD refers to the death of tumour cells after PDT, which stimulates the immune system to produce a strong immune response by releasing damage-associated molecular patterns (DAMPs), cytokines, tumour-associated antigens (TAA) and other signalling molecules (279). These DAMPs can be recognized by the immune system and activate antitumour immune responses. DAMPs mainly include calreticulin (CALR), heat shock proteins 70/90 (HSP70/90), ATP, high-mobility group box-1 (HMGB1) nuclear protein, type I interferons (IFNs) and members of the IL-1 cytokine family, etc. In addition, ROS produced by PDT can destroy tumour blood vessels, limit tumour nutrient supply, and stimulate antitumour immune responses (262). Cytokines are able to trigger an inflammatory response that further enhances immune cell infiltration and activation (280, 281). The mechanism of PDIT in gliomas is illustrated in **Figure 2**.

#### 4.1.2 SDT

Ultrasound (US) is a kind of mechanical vibration wave with strong tissue penetration ability that has been widely used in ultrasound imaging and ultrasound therapy. Among the US-derived techniques, SDT based on ROS production is a good strategy. Research on SDT began in the 1990s (289, 290). Based on the bibliometric analysis of SDT, studies have shown that since 2000, SDT has experienced rapid growth and has mainly focused on the fields of nanomaterials and cancer treatment, achieving significant results (291). The mechanism of SDT is to use low-frequency ultrasound to trigger sonosensitizers that accumulate at the tumour site, producing ROS and cavitation bubbles to kill the tumours. These ROS produce significant toxic effects on tumour cells in the 1 μm range (292, 293). The advantage of SDT is mainly that ultrasound can penetrate to a depth of 10 cm, which can kill tumours in deeper locations (294, 295). At present, most of the sonosensitizers used in reported SDT are photosensitizers or are derived from photosensitizers (296). However, SDT also has difficulty achieving the ideal tumour killing effect due to the presence of the BBB and the poor targeting effect of sonosensitizers such as porphyrins (297). Notably, TMZ can not only penetrate the BBB but also act as a sonosensitizer to induce necroptosis in GBMs. This provides new potential options for treating GBMs with SDT (293).

According to the literature, although SDT has been studied in gliomas for less than 20 years, it has shown promising efficacy in preclinical studies (30, 298, 299). However, due to the maturity of the technology and the factors of ultrasound equipment and other objective reasons, the research results of SDT are less than those of PDT, and clinical research is also in its infancy. At present, there are three clinical trials of SDT in gliomas under recruitment (based on clinicaltrials.gov), as shown in **Table 2**. More clinical trials are needed to verify the efficacy of SDT in the glioma treatment.

#### 4.1.3 CDT

The concept of CDT was first proposed in 2016 by Bu, Shi et al. (300). CDT is dependent on transition metal ions in the TME to produce high levels of OH· through Fenton/Fenton-like reactions, resulting in tumour killing (301). The Fenton reaction refers to the complex chemical reaction of ferrous ions with H_2_O_2_, which eventually generates highly toxic OH· (302). Catalysts for Fenton-like reactions are usually other transition metals, such as copper (Cu) and manganese (Mn) (303, 304). Compared with PDT, the advantage of CDT is that it does not require laser irradiation, so it can avoid the limitations caused by light penetrating the tissues. Alternatively, the TME is characterized by acidity and H_2_O_2_ overexpression, which favours Fenton/Fenton-like reactions. However, when the pH at the tumour site is too high or H_2_O_2_ production within the tumour is insufficient, the Fenton/Fenton-like reaction will be insensitive, and CDT efficiency will be reduced (305). In general, CDT has the advantages of strong targeting, low adverse reactions, regulation of TME hypoxia, and low treatment cost, so it has great potential to be used in tumour therapy (301). In the glioma treatment, CDT is still in the preclinical stage, and no clinical trials have been carried out. However, it has been shown that CDT has good therapeutic efficacy and can exert more anti-glioma effects in combination with PDT (306) and photothermal therapy (PTT) (307).

#### 4.1.4 Breaking through the BBB to enhance PDT/SDT/CDT

The BBB is a physical, chemical and biological barrier structure formed by capillary endothelial cells in the brain, surrounding astrocytes and muscle rings. The main function of the BBB is to maintain the stability of the brain environment, regulate the entry of nutrients, and prevent harmful substances from entering the brain through the blood (308, 309). Brain endothelial cells are composed of hydrophobic lipid bilayers with tight junctions. Therefore, drugs with large polarity and molecular weight often have difficulty passing the BBB (310). However, research has shown that human glioma cells can infiltrate through the perivascular space and extensively invade the brain away from the tumour mass. In this process, glioma cells displace the end feet of astrocytes, thereby disrupting the BBB, which may be beneficial for drug therapy (311, 312). However, effectively overcoming the limitations of the BBB remains a challenge. Currently, nanodrug delivery platforms (313), microbubble-enhanced focused ultrasound (MB-FUS) (314) and magnetic resonance-guided focused ultrasound (MRg-FUS) (315) are three promising approaches to break through the BBB.

Nanotechnology is the study and application of particles or structures between 1 and 100 nm, where it can maximize drug transport and targeted delivery (316–318). Nanodrug delivery platforms are the application of nanotechnology in medicine. In general, nanodrug delivery platforms are usually composed of nanocores, nanocarriers, targeting ligands, drugs and surface modifications or may not completely contain these parts. Among them, the nanocore is the main component of the platform, mainly serving to support and stabilize the nanodrug delivery platform. It can be composed of materials such as gold (319), silicon (320), magnetic materials (321), etc. Nanocarriers refer to carriers that carry drugs on nanocores. An ideal nanocarrier can stably encapsulate drugs inside and release them at the appropriate time (322). Types of nanocarriers include gold nanoparticles, magnetic nanoparticles, carbon nanotubes, polymer micelles, and liposomes (323), etc. Targeting ligands attached to the nanocore include antibodies (324) and targeting peptides (325), which can precisely target the target. Nanocarriers can carry drugs, which include PSs such as chlorin e6 (Ce6) (326), immune checkpoint inhibitors such as nivolumab (327), and chemotherapy drugs such as doxorubicin (DOX) (328). Surface modification refers to the modification of the surface of the nanoplatform, such as polyethylene glycol (PEG), to enhance the hydropathy, stability, and biocompatibility of the nanoplatform and improve the retention time *in vivo* (329).

In general, a well-functioning nanodrug delivery platform can typically enhance the therapeutic effects of PDT (330), SDT (331), and CDT (332) for the treatment of glioma. Moreover, optimizing key components of nanodrug delivery platforms can be an effective strategy to break through the BBB. First, the targeting of the nanoplatform should be enhanced. Transferrin (TF), for example, targets a transferrin receptor (TFR) that is overexpressed on the surface of brain capillary endothelial cells and malignant brain tumours. Related studies have shown that TF-bound nanoplatforms can effectively cross the BBB and target gliomas (333, 334). Second, the development of new nanocarriers, such as nanotubes, gold nanoparticles, and magnetic nanoparticles, makes it easier for drugs to reach the glioma site (335–337). Furthermore, polymers such as polyethylene glycol (PEG) and polylactic-co-glycolic acid (PLGA) can be used to encapsulate the drug to allow crossing of the BBB. The advantages of such polymers are good biocompatibility, easy surface modification, etc., and the ability to control the rate of drug release (329, 338). When a nanodrug delivery platform is combined with PDT, SDT, and CDT, it will greatly increase the targeting ability of the three therapeutic strategies and the level of ROS production, leading to a “1 + 1 > 2” therapeutic effect. The summary of glioma-related research on the nanodrug delivery platforms combined with PDT, SDT, and CDT is shown in **Table 3**.

**Table 3:** Summary of nanodrug delivery platforms used for glioma PDT/SDT/CDT.

Categories	Nanocarriers	Drugs	Targeting ligands	Target receptors	Results	References
**PDT**	MRN	[Ru(bpy)_2_(tip)]^2+^	TF, aptamer AS1411	TFR, nucleolin	The glioma cells undergo apoptosis.	(334)
	nanotube	IR780, DOX	———	———	The glioma cells undergo apoptosis.	(335)
	AuNs	ICG, DOX	TF	TFR	It effectively kills glioma cells.	(336)
	GuIX nanoparticle	porphyrin molecule	KDKPPR	NRP-1	Glioma vessels are occluded, causing growth delay.	(337)
	polyethylene glycol	IR780, camptothecin	iRGD peptide	αvβ3/5, NRP-1	The glioma cells undergo apoptosis.	(339)
	albumin	CAP	_____	_____	The glioma cells undergo apoptosis and necrosis.	(340)
	lipidosome	Ce6, LOMs	_____	_____	The generation of a large amount of O_2_ promotes the effect of PDT in the glioma treatment.	(341)
	BN	Ce6, DOX	platelet	tumour vascular endothelium	A large amount of ROS is generated, which subsequently leads to the death of glioma cells.	(342)
	FLs	ICG, DOX	G-Anti G	neuropilin-1 on glioma cells	The production of ROS leads to the death of the majority of glioma cells.	(325)
**SDT**	liposome	sinoporphyrin sodium	iRGD peptide	αvβ3/5, NRP-1	SDT enhances its effect by increasing the production of a significant quantity of ROS, leading to the subsequent death of glioma cells.	(331)
	liposome	Ce6, HCQ	ANG-2	LRP1	Nanosensitizers have a strong response to ultrasound, leading to the generation of a large amount of ROS and subsequently causing glioma cell apoptosis.	(343)
	SiO_2_ nanoparticle	CAT, ICG	aptamer AS1411	nucleolin	CAT catalyzes the generation of O_2_ from H_2_O_2_, alleviating tumour hypoxia and improving the efficiency of SDT.	(344)
	nanocrystal	ppIX, MnO2	holo-TF	TFR	O_2_ is produced in large quantities and Mn^2+^ is released to achieve efficient SDT.	(345)
**CDT**	polymeric micelle	Mn^2+^, TMZ	iRGD	*α*v*β*3 integrin, NRP-1	TMZ、Mn^2+^and O_2_ release. Subsequently, glioma cells undergo death.	(346)
	Hb@GOx nanoparticle	Hb, GOx	_____	_____	The levels of H_2_O_2_ and OH**·** increase in glioma cells. Subsequently, glioma cells undergo death.	(253)
	liposome	Ce6, SPIOCs	_____	_____	The levels of H_2_O_2_ and OH**·** increase in glioma cells. Glioma cells subsequently die.	(347)

MRN, mesoporous ruthenium nanoparticles; AuNs, gold nanoparticles; ICG, indocyanine green; DOX, doxorubicin; TF(R), transferrin (receptor); NRP-1, neuropilin-1; αvβ3/5, integrin alpha V beta 3/5; CAP , chloro-aluminiumphtalocyanine; LOMs, lipid-coated oxygen microbubbles; BN, boron nitride; FLs, fluorescent poly(levodopamine) nanoparticles; HCQ, hydroxychloroquine; ANG-2, angiopep-2; LRP1, low density lipoprotein receptor-related protein 1; ppIX, protoporphyrin; holo-TF, holo-transferrin; Hb, hemoglobin; Gox, glucose oxidase; SPIOCs, superparamagnetic iron oxide nanoclusters

In addition to nanodrug delivery platforms, the use of MB-FUS (348–350) and MRg-FUS (315) to open the BBB for drug delivery both has great potential. Microbubbles are essentially small bubbles of biocompatible gases, such as nitrogen or perfluorocarbon, encapsulated in a lipid, protein, or polymer membrane (351). As blood with microbubbles flows through the brain, ultrasound waves are emitted precisely to target areas. The ultrasound stimulates microbubbles to oscillate violently and burst, producing a temporary, local pressure change that can temporarily open the tight junctions of the BBB and increase its permeability. In this way, drugs or macromolecules that cannot penetrate the BBB can enter the brain tissues, thus allowing for the effective treatment of gliomas (343, 352, 353). In recent studies, MB-FUS achieved BBB opening and increased drug aggregation in GBM regions to enhance antitumour effects (354–357). Notably, the method of opening the BBB using ultrasound microbubbles is reversible, does not damage neurons, and the BBB heals a few hours after exposure (358). Thus, this method has great potential for application. This technique is still in its early stages, and promising results have been demonstrated in clinical trials in glioma patients (359). Furthermore, as another method to open the BBB, MRg-FUS can accurately focus ultrasonic waves on the GBM region and provide real-time monitoring and guidance during therapy provided through magnetic resonance imaging (MRI). Ultrasound energy can raise the temperature of the BBB region, thus enhancing permeability. Consequently, platinum nanoparticles can more effectively penetrate the GBM tissues, thereby inhibiting the growth of GBM cells (315).

### 4.2 Treatment to scavenge/reduce ROS levels

Antioxidants are a class of compounds that inhibit oxidation by scavenging ROS and reducing OS, and they can help reduce or block oxidative reactions in cells (360). When OS occurs, antioxidants interact with ROS to capture and neutralize ROS, thereby protecting cells from oxidative damage (361). Common antioxidants include vitamin C, vitamin E, α-carotene, selenium, etc., which can be obtained through food intake or supplements (362). Furthermore, the use of antioxidants can inhibit tumorigenesis by preventing OS caused by various causes, and the mechanism is to repair damaged DNA and inhibit cancer occurrence, including gene mutations, oxidative chromosomal damage, and lipid peroxidation of cell membranes (131, 363).

There is considerable evidence that intake of antioxidants may help reduce the risk of gliomas (364). For example, CoQ10 can act as a ROS scavenger to increase the sensitivity of gliomas to TMZ, thereby inhibiting the invasion of glioma cells *in vitro* and *in vivo*. Mechanistically, CoQ10 can integrate into the mitochondrial membrane and reduce ROS production. It also reduced the expression of MMP9 and epithelial-mesenchymal transition (EMT) markers (28, 365). Naringenin is an antioxidant. Naringenin supplementation for 1 month can reduce lipid peroxidation and decrease the expression of PKC, NF-κB, cyclin D1(CCND1) and cyclin-dependent kinase 4 (CDK4), thereby inhibiting the proliferation of glioma cells in mouse models (27). Astaxanthin is a natural carotenoid, and adonixanthin is a product of its formation (366). Studies have confirmed that both have strong antioxidant capacity, which can cross the BBB and protect brain tissues from ischaemia or hemorrhage (367, 368). In mouse glioma models, astaxanthin and adonixanthin intake increased p38 phosphorylation in glioma cells, leading to cell cycle arrest. Furthermore, adonixanthin was able to reduce the expression of MMP2 and fibronectin downstream of ERK1/2 and AKT signalling pathways and inhibit invasion and metastasis in both *in vitro* and *in vivo* GBM models (369). Chrysin is a kind of flavonoid with antioxidant properties. The p38-MAPK pathway is activated in rat glioma cells treated with chrysin, resulting in the accumulation of p21 (WAF1/CIP1) protein, decreased activities of CDK2 and CDK4, and cell cycle arrest in G1 phase (32). Similarly, hypoxia-inducible factor-1alpha (HIF-1α) expression was blocked when the antioxidant melatonin was used, resulting in a significant inhibition of MMP2 and VEGF expression, thereby inhibiting GBM cell migration and invasion *in vitro* (370). Moreover, antioxidants quercetin (QE), baicalein (BE) and myricetin (ME) effectively downregulated ROS and MMP9 and inhibited glioma cell invasion/migration events *in vitro* (24).

However, some studies have shown that intake of antioxidants, such as carotenoids (371), vitamin E (372), and coffee (373), is not associated with the risk of developing gliomas. This may be related to factors such as bioavailability, dose, BBB permeability, and tumour heterogeneity (364). Therefore, the role of ROS scavenging using antioxidants in glioma therapy still needs to be confirmed by more studies.

## 5 Conclusion

ROS are products of cellular redox and play important functions in cells. Excessively high or low ROS levels are detrimental to cell survival. That is, there is a threshold for intracellular ROS, and when a large accumulation of ROS exceeds the threshold and cannot be neutralized by the antioxidant defence system, it leads to OS and thus cell death. For gliomas, there is also a threshold for ROS. Appropriate ROS levels can aid survival, but high levels of ROS can also lead to their own death. Therefore, ROS-based therapies are particularly important.

Currently, there are two common therapeutic approaches involving ROS in the therapy of glioma, which are increasing ROS levels to induce cell death or using antioxidants to inhibit progression. In terms of increasing ROS, PDT/SDT/CDT is the representative approach. With the development of modern nanotechnology, the corresponding drugs can better pass through the BBB. Preclinical studies have shown that PDT/SDT/CDT combined with nanotechnology shows potent antiglioma effects and has good potential for clinical application. Conversely, ROS reduction using antioxidants has also been shown to inhibit glioma initiation and progression. Nonetheless, both strategies have limitations. In addition to the unclear clinical efficacy of antioxidants for cancer treatment reported in the literature, there are also issues such as the uncertain toxicity and biosafety of nanomaterials (374, 375), and the uncertain stability and retention time of nanodrug delivery platforms (376). Therefore, in the future development of nanodrug delivery platforms targeting gliomas, it is necessary to enhance the targeting and stability of nanoparticles and improve the ability to cross the BBB and biosafety to provide effective treatment while reducing adverse reactions. At present, although there are still many obstacles to ROS-based therapy, ROS still have the potential to be widely used as a therapeutic target for gliomas.

## Author contributions

Y-CY: Writing – original draft. YZ: Writing – original draft. S-JS: Visualization, Writing – original draft. C-JZ: Writing – review & editing. YB: Writing – review & editing. JW: Writing – review & editing. L-TM: Supervision, Writing – original draft, Writing – review & editing.
